# Breast milk miRNAs and their potential role in the development of atopy in infants

**DOI:** 10.3389/fmolb.2026.1768239

**Published:** 2026-04-01

**Authors:** Adrianna Porębska, Maciej Maj, Aizhan Rakhmetullina, Piotr Zielenkiewicz, Leszek Pączek

**Affiliations:** 1 Department of Clinical Immunology, Medical University of Warsaw, Warsaw, Poland; 2 Institute of Biochemistry and Biophysics, Polish Academy of Sciences, Warsaw, Poland; 3 Institute of Experimental Plant Biology and Biotechnology, University of Warsaw, Warsaw, Poland

**Keywords:** asthma, atopic dermatitis, atopy, breastfeeding, human milk, immune development, miRNA

## Abstract

Human breast milk is a dynamic biological fluid enriched with bioactive components, including extracellular vesicle-derived microRNAs (miRNAs), which have emerged as potential mediators of early immune programming. Growing evidence suggests that maternal atopic status can alter the breast milk miRNA profile, potentially shaping the infant’s susceptibility to atopic disorders, such as atopic dermatitis, asthma, and food allergy. This review presents current research examining the link between breast milk miRNA composition and the development of atopy in infants, with a particular focus on maternal atopic status. Four studies met eligibility criteria and collectively demonstrate that maternal conditions such as asthma or atopic dermatitis are associated with distinct breast milk miRNA signatures. Certain miRNAs, e.g., miR-375-3p and miR-1290, show altered expression in the milk of atopic mothers. Elevated levels of miR-375-3p are associated with a reduced risk of infant atopic manifestations during the first year of life, including atopic dermatitis, food allergy, and wheezing. Conversely, miR-1290 is significantly upregulated in the milk of mothers with asthma and atopy during pregnancy, even after adjusting for confounders, highlighting its potential role as a biomarker of maternal allergic status. However, current findings rely primarily on statistical associations; no human study has yet demonstrated direct transfer or functional activity of milk-derived miRNAs in infants. Overall, while breast milk miRNAs represent a promising link between maternal health and infant atopy risk, conclusive evidence is lacking. Future large-scale, standardized, longitudinal studies integrating functional validation are needed to clarify mechanistic pathways and evaluate the potential of milk miRNAs as biomarkers or targets for early atopy prevention. Understanding the functional impact of breast milk miRNAs could facilitate the development of non-invasive biomarkers for predicting atopy risk, as well as early-life preventive strategies.

## 1 Introduction

Human breast milk is a dynamic biological fluid that provides not only essential nutrients, such as lactose, lipids, and proteins, but also a wide variety of bioactive factors, including hormones, lactoferrin, human milk oligosaccharides, cytokines, and immunoglobulins (Brockway et al., 2024). These molecules are believed to offer passive protection against pathogens, support maturation of the infant immune system, and reduce the risk of developing allergic and autoimmune disorders in the future (Brockway et al., 2024; Masi and Stewart, 2024). Among these bioactive components, extracellular vesicles (EVs) have recently been recognized as potentially important mediators of mother–infant communication. These nano-sized lipid-bound structures can facilitate intercellular communication by transporting proteins, lipids, and nucleic acids, including microRNAs (miRNAs) (Golan-Gerstl and Reif, 2022), and are thought to serve as carriers of maternal signals to the infant.

miRNAs are short (18–24 nucleotides), single-stranded, non-coding RNAs that regulate gene expression (O’Brien et al., 2018). They are involved in key biological processes, including embryonic development (Gross et al., 2017), tissue differentiation (Saliminejad et al., 2019), and the regulation of cell proliferation and apoptosis (Annese et al., 2020). The discovery of these molecules, for which Victor Ambros and Gary Ruvkun were awarded the 2024 Nobel Prize, revolutionized molecular biology and initiated a rapidly developing field of study (Callaway and Sanderson, 2024).

Notably, substantial amounts of miRNA are packaged within breast milk EVs (Jiang et al., 2021). Many of these miRNAs are highly conserved across species (Herwijnen et al., 2018), which may suggest an important evolutionary role.

These characteristics raise questions about their physiological significance, particularly in early immune development. This could influence the risk of, e.g., atopy, which is characterized by allergen sensitization and a Th2-dominated immune phenotype, along with impaired regulatory T-cell (Treg) function and epithelial barrier defects (Conrad et al., 2025). These processes promote chronic inflammation and increase the risk of developing atopic diseases–including atopic dermatitis, food allergy, or asthma (Conrad et al., 2025). Understanding the development of allergic diseases is crucial, as these conditions are a growing global concern due to their high prevalence. For instance, atopic dermatitis affects approximately 4% of pediatric patients worldwide. In some regions, however, its occurrence can even reach 10%–20% (Tian et al., 2023).

Milk-derived miRNAs could play a role in the complex pathophysiology of allergic diseases. Experimental and observational studies have shown that these miRNAs can survive digestion, cross the intestinal barrier, and reach different organs, including the thymus and spleen (Tian et al., 2023; Xu et al., 2024). In various organs and tissues, they modulate infant immunity (Ahlberg et al., 2023a; Ahlberg et al., 2023b) and metabolic pathways (Alonso-Bernáldez et al., 2022) relevant to the development of allergic diseases. Moreover, multiple miRNAs have been identified to influence Treg development (Melnik et al., 2014), target immune-related pathways along with cytokine production (Pua et al., 2016; Arenas-Padilla and Mata-Haro, 2018), and promote the development of oral tolerance to ingested antigens (Ahlberg et al., 2023a). All of these processes play critical roles in atopy prevention.

Interestingly, maternal factors, such as diet (Hicks et al., 2022a), infection (Alsaweed et al., 2025), and illnesses, like asthma (Bozack et al., 2022) or obesity (Shah et al., 2021), can significantly alter the composition of breast milk miRNAs. Current evidence demonstrates that miRNAs are dysregulated in atopy (Yu et al., 2020) and are present in human milk (Kosaka et al., 2010). Although the exact mechanisms linking maternal atopy to infant atopy risk remain poorly defined, growing evidence suggests that the presence of maternal atopy may influence infant susceptibility through alterations in breast milk miRNA concentrations (Bozack et al., 2022).

The aim of this review is to summarize current knowledge on alterations in the breast milk miRNA profile in relation to atopy in offspring and mothers. Existing challenges in the field are highlighted, and potential miRNA-mediated pathways involved in the pathophysiology of atopic diseases are discussed.

## 2 Search strategy and study selection

This review was conducted as a systematic narrative review, combining a structured literature search with a descriptive synthesis of the available data in order to examine the relationship between breast milk miRNA profiles and atopy. A comprehensive search, in accordance with PRISMA guidelines, was performed using the electronic bibliographic databases MEDLINE, Scopus, and Web of Science, focusing on publications between January 2010 and August 2025. The search strategy was based on terminology and keywords found in articles on miRNA, human milk, and atopy. Boolean operators (“OR”, “AND”) were used to combine the search terms listed below within and between concepts. The search string consisted of three conceptual domains.

- miRNA domain - (“microRNA” OR “miRNA”)
- human milk domain - (“milk” OR “human milk” OR “breastmilk”)
- atopy condition domain - (“atopy” OR “atopic” OR “allergy” OR “eczema”)

This search strategy aimed to maximize the sensitivity in identifying relevant records while preserving specificity to the research question.

Studies were included if they:

- investigated human breast milk samples;
- analyzed miRNA expression/profiles;
- compared mothers with atopic conditions to healthy controls;
- reported original data (case reports, observational studies, cohort studies, randomized trials);
- were published between January 2010 and August 2025;
- were indexed in MEDLINE, Scopus, and/or Web of Science;
- were in English and were available in full-text form.

Studies were excluded if they:

- did not investigate human breast milk miRNA profiles in relation to the presence of an atopic condition;
- were based only on *in vitro* or animal studies;
- were reviews, meta-analyses, conference abstracts, editorials, or commentaries;
- were published before January 2010;
- were not available in full-text form or were in a language other than English.

**FIGURE 1:**
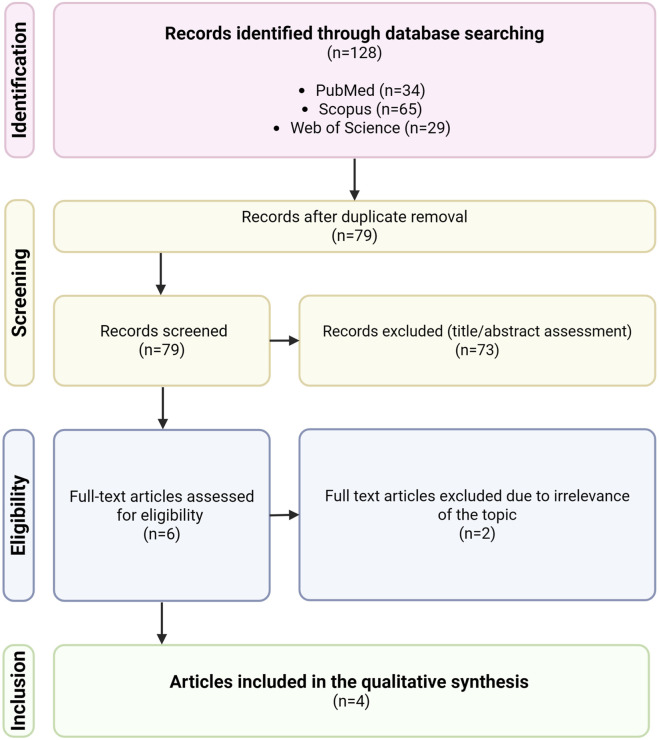
Flow diagram illustrating the literature search strategy. Created in BioRender. Maj, M. (https://BioRender.com/rytv2pi).

The literature search process is summarized in Figure 1. A total of 128 records were identified through database searching. After 49 duplicates were removed, the titles and abstracts were screened. Seventy-three records were excluded for irrelevance, as these studies did not investigate human milk miRNA profiles in mothers with atopy. Of the remaining six articles, a full-text review revealed four studies that met the inclusion criteria and provided relevant data on the impact of maternal atopy on milk miRNA composition.

## 3 Human breast milk miRNAs and atopy: from biogenesis to clinical evidence

### 3.1 Biogenesis and functional roles of breast milk miRNAs

**FIGURE 2:**
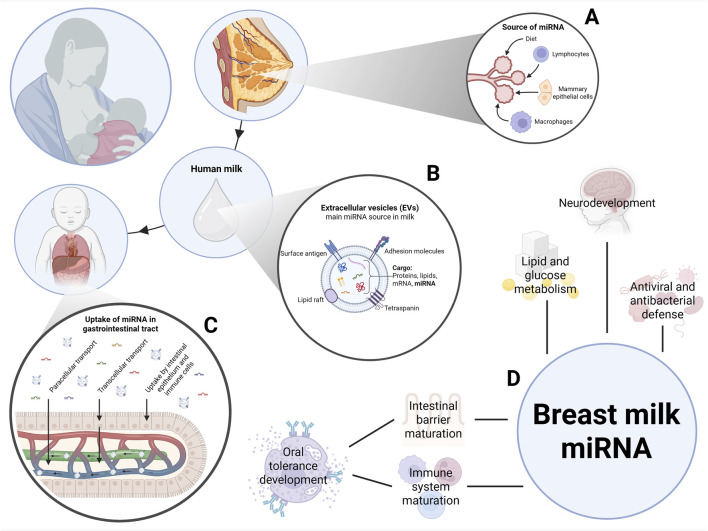
Overview of the biology and functions of breast milk–derived miRNAs. **(A)** Sources of miRNAs in human milk, including immune cells, mammary epithelial cells, and maternal diet. **(B)** Structure and cargo of extracellular vesicles (EVs) present in milk and involved in miRNA transport. **(C)** Postulated mechanisms of milk-derived miRNA absorption in the infant digestive tract. **(D)** Potential biological functions of breast milk-derived miRNAs. Created in BioRender. Maj, M. (https://BioRender.com/fgpgnb6).

Human milk miRNAs originate primarily from mammary epithelial cells. However, immune cells, including macrophages and lymphocytes, may also secrete miRNAs into the milk (Figure 2A) (Alsaweed et al., 2016). Although milk miRNAs exist in three main physical states–enclosed within EVs, bound to proteins or lipoproteins, or as unencapsulated (“free”) miRNA–the primary miRNA source bioavailable to the offspring appears to be encapsulated within EVs (Figure 2B) (Zhou et al., 2012; Carrillo-Lozano et al., 2020).

The packaging of nucleic acids, including miRNAs, into human milk EVs is a highly regulated, multi-step process originating within mammary epithelial cells. These EVs function as a delivery system, transferring maternal signals to the infant (Husseini and Gilbert, 2025; Admyre et al., 2007). This complex process ensures that the RNA molecules are safely encapsulated within the durable lipid bilayer, protecting them from degradation in the mother’s milk and infant’s digestive tract (Yung et al., 2024; Liao et al., 2017).

Orally delivered EVs have been shown to be taken up by intestinal cells (Weil et al., 2023). Uptake mechanisms include primarily transcellular transport, such as receptor-mediated endocytosis by epithelial cells, and paracellular passage (Figure 2C) (Aarts et al., 2021). The intestinal uptake of milk-derived miRNAs may influence infant development and immunity (Figure 2D) (Jiang et al., 2021; Xu et al., 2024). Experimental studies have shown that milk miRNAs can modulate the development of oral tolerance (Ahlberg et al., 2023a). Evidence indicates that milk-derived miRNAs transferred via EVs, including miR-155, promote Treg differentiation (Melnik et al., 2014; Admyre et al., 2007), which is crucial for immune tolerance (Sakaguchi et al., 2008). By promoting Tregs, milk miRNAs help downregulate IL-4/Th2-mediated atopic sensitization (Melnik et al., 2014), thereby suppressing immune pathways involved in allergy pathophysiology (Shankar et al., 2022).

However, human studies on this topic remain limited. In contrast, a substantial body of evidence from *in vitro* and animal models has explored the role of human milk-derived miRNAs in atopy. For instance, Liu et al. demonstrated that administration of EVs carrying miR-146a-5p to mice with induced asthma reduced airway inflammation and Th2-type allergic markers (Liu et al., 2024). Similarly, treatment with an antagomir targeting miR-126 in a mouse model of allergic airway disease markedly suppressed the Th2-type response (Mattes et al., 2009), a key driver of atopic inflammation (Douglass and O’Hehir, 2006; Contreras et al., 2003). Together, these findings indicate that milk miRNAs may attenuate type 2 inflammatory responses associated with atopy. Nevertheless, clinical studies are required to confirm these effects in infants.

### 3.2 The pathophysiology and clinical manifestations of atopy

Allergic diseases develop when the immune system elicits a hypersensitive response to normally harmless environmental substances, known as allergens (Douglass and O’Hehir, 2006). Some individuals, particularly children, have a family tendency to develop this excessive immune response–a trait called atopy (Douglass and O’Hehir, 2006). Following exposure to common allergens such as dust, pollen, or food, the offspring’s immune system drives production of IgE antibodies and activates a Th2-type response characterized by cytokines including IL-4 and IL-13 (Contreras et al., 2003). This atopic tendency often manifests as atopic dermatitis (eczema), allergic rhinitis (hay fever), and asthma (Dreborg, 2002). Atopic dermatitis is characterized by recurrent skin inflammation, impaired barrier function, and elevated IgE levels (Leung et al., 2004), while asthma is a chronic inflammatory disease of the airways marked by reversible airflow obstruction, mucus production, and bronchial hyperreactivity often triggered by allergens (Murdoch and Lloyd, 2010). These interconnected atopic diseases frequently occur together and can significantly reduce a child’s wellbeing and health (Hill and Spergel, 2018).

### 3.3 Human milk miRNAs and infant atopy: current evidence

The relationship between breastfeeding and atopy in offspring remains controversial, yet emerging evidence suggests that human milk may influence the development of atopy in infants. The four studies described in this review provide complementary insights into this topic. Simpson et al. were among the first to investigate this potential influence, examining whether specific milk miRNA profiles correlated with the development of atopic dermatitis in children (Simpson et al., 2015). The scientists identified a stable core group of highly expressed miRNAs in breast milk, including miR-148a-3p and miR-22-3p (Simpson et al., 2015). Based on *in silico* analyses, they were predicted to influence a broad spectrum of biological processes (Simpson et al., 2015), though their specific effects still need further examination.

In a different approach, Bozack et al. performed an observational study focusing on maternal physiology, analyzing how conditions such as asthma or atopy alter the composition of milk-derived EVs without directly assessing infant outcomes (Bozack et al., 2022). The authors found that specific miRNAs in breast milk-derived EVs, including miR-1290, were associated with these maternal conditions and may influence infant immune development (Bozack et al., 2022). A bioinformatic pathway analysis performed in this study predicts a potential role for these miRNAs in immune regulation through mechanisms such as TGF-β signaling and extracellular matrix–receptor interactions. Their exact effect on infant atopy development still requires laboratory confirmation though (Bozack et al., 2022).

Moving beyond compositional analysis to clinical observations, Hicks et al. addressed the gap between milk composition and infant health by estimating the total daily intake of specific miRNAs present in milk lipids and relating them to clinical infant outcomes (Hicks et al., 2022b). The study demonstrated that higher levels of miR-375 in human milk lipids were associated with a lower risk of atopic diseases in infants (Hicks et al., 2022b). While the exact mechanisms need to be fully elucidated, the study suggested that miR-375 may influence immune system development in infants, potentially by modulating gene expression related to immune responses (Hicks et al., 2022b).

Finally, Ahlberg et al. explored the immunological mechanism, finding that certain breast milk miRNAs correlate with higher levels of Tregs in infants (Ahlberg et al., 2023b). Given the crucial role of Tregs in immune tolerance, these findings suggest that milk miRNAs may support the regulation of allergic responses (Ahlberg et al., 2023a; Ahlberg et al., 2023b). Nevertheless, a causal link cannot be established as this study did not quantify direct miRNA transfer to the infant (Ahlberg et al., 2023b). The authors found that in breastfed children, miR-148a-3p, let-7d-3p, miR-181a-3p and miR-181c-3p correlated with higher levels of activated Treg cells in infants (Ahlberg et al., 2023b). However, among these, only the let-7 family has been consistently confirmed to be altered in the context of atopy (Ahlberg et al., 2023a).

Together, these findings highlight how maternal health and milk composition shape infant atopy risk. Among the diverse bioactive components, milk miRNAs have emerged as a promising link between maternal factors and infant susceptibility to allergic disease.

To provide a comprehensive overview of these molecular associations, Table 1 summarizes available data on breast milk miRNA concentration changes observed in various maternal atopic conditions and further outlines immunomodulatory genes and pathways targeted by each miRNA within the context of atopy and allergy research. Only experimentally validated miRNA targets associated with atopy were included.

**TABLE 1:** Differentially expressed miRNAs in atopic conditions and their reported gene targets.

No.	miRNA	Up/Downregulation	Analysed conditions	Atopy related miRNA targets	References
1	miR-148b-3p	Up	Atopic dermatitis, food allergies, wheezing, inactive asthma	*HLA-G* (Tan et al., 2007) - asthma	Bozack et al. (2022) and Hicks et al. (2022b)
2	miR-148b-3p	Down	Active asthma during pregnancy	—	Bozack et al. (2022)
3	miR-375-3p	Up	Atopic dermatitis, food allergies, wheezing	*YAP1* (Cheng et al., 2020) – atopic dermatitis *TSLP* (indirect positive regulation) (Luo et al., 2020) – allergic rhinitis *KLF4* (Wang et al., 2020) – allergic rhinitis *JAK2* (Wang et al., 2018) – allergic rhinitis	Hicks et al. (2022b)
4	miR-146b-5p	Up	Atopic dermatitis	—	Simpson et al. (2015)
5	miR-21-5p	Up	Atopic dermatitis	*Smad7* (Hur et al., 2021) – airway inflammation *IL-12p35, IL-12* (Lu et al., 2009; Lee et al., 2021)– asthma *PARP-1* (Mao et al., 2024) – asthma, allergic rhinitis *DDAH1* (Zou et al., 2021) – asthma	Simpson et al. (2015)
6	miR-22-3p	Up	Atopic dermatitis	*NLRP3* (Guo et al., 2021) – asthma *CBL, ESR1* (Dong et al., 2016) – children with dust mite-induced asthma	Simpson et al. (2015)
7	let-7f-5p	Up	Atopic dermatitis	*IL-23R* (Newcomb et al., 2015) – severe asthma	Simpson et al. (2015)
8	let-7d-3p	Up	Atopic dermatitis	—	Simpson et al. (2015)
9	miR-511-5p	Up	Atopic dermatitis	—	Simpson et al. (2015)
10	miR-26b-5p	Up	Atopic dermatitis	—	Simpson et al. (2015)
11	miR-30e-5p	Up	Atopic dermatitis	—	Simpson et al. (2015)
12	miR-335-5p	Up	Atopic dermatitis	*SOX6* (Liew et al., 2020) – atopic dermatitis	Simpson et al. (2015)
13	miR-1290	Up	Active asthma and general atopy during pregnancy	—	Bozack et al. (2022)
14	miR-191-5p	Down	Active asthma during pregnancy	—	Bozack et al. (2022)
15	miR-200a-3p	Down	Active asthma during pregnancy	—	Bozack et al. (2022)
16	miR-324-5p	Down	Active asthma during pregnancy	—	Bozack et al. (2022)
17	miR-324-5p	Up	Inactive asthma during pregnancy	—	Bozack et al. (2022)
18	miR-29a-5p	Down	Active asthma during pregnancy	*FOS* (Fan et al., 2021) – allergic rhinitis	Bozack et al. (2022)
19	miR-331-3p	Down	Active asthma during pregnancy	—	Bozack et al. (2022)
20	miR-30a-3p	Down	Active asthma during pregnancy	*CCR3* (Li et al., 2020a) – asthma *RUNX2/HMGB1* (Wu et al., 2022) – asthma	Bozack et al. (2022)
21	miR-30a-3p	Up	Inactive asthma during pregnancy	—	Bozack et al. (2022)
22	miR-106b-5p	Down	Active asthma during pregnancy	*LIF* (Li et al., 2021) – allergic rhinitis *Erg-2* (Tang et al., 2015) – allergic rhinitis *SIX1* (Liu et al., 2021) – asthma	Bozack et al. (2022)
23	miR-193a-5p	Up	Inactive asthma and atopy during pregnancy	*IL-4* (D’Argenio et al., 2018) – cow’s milk allergy	Bozack et al. (2022)
24	miR-224-5p	Up	Inactive asthma and atopy during pregnancy	*GATA3* (Li et al., 2024) – allergic rhinitis *TLR4* (Wu et al., 2021) – allergic rhinitis *CDK9* (Wang et al., 2021) – allergic rhinitis *FHL1* (Zhuang et al., 2022) – asthma *TLR2* (Li et al., 2020b) – asthma *CLDN5* (Yang et al., 2024) – skin permeability	Bozack et al. (2022)
25	miR-24-3p	Up	Inactive asthma and atopy during pregnancy	—	Bozack et al. (2022)
26	miR-218-5p	Up	Inactive asthma and atopy during pregnancy	*CTNND2* (Liang et al., 2020) – eosinophilic airway inflammation	Bozack et al. (2022)
27	miR-19b-3p	Up	Inactive asthma and atopy during pregnancy	*SOCS-1* (Kang et al., 2025) – allergic rhinitis	Bozack et al. (2022)
28	let-7e-3p	Down	Maternal allergic diseases	—	Ahlberg et al. (2023b)

*CBL* (Casitas B-lineage lymphoma proto-oncogene); *CCR3* (C-C chemokine receptor type 3); CDK9 (cyclin-dependent kinase 9); *CLDN5* (claudin 5); *CTNND2* (catenin delta 2); *DDAH1* (dimethylarginine dimethylaminohydrolase 1); *Erg-2* (ETS-related gene 2); *ESR1* (estrogen receptor 1); *FHL1* (four and a half LIM domains 1); *FOS* (*Fos Proto-Oncogene*); *GATA3* (GATA binding protein 3); *HLA-G* (human leukocyte antigen G); *IL-12* (interleukin 12); *IL-12p35* (interleukin-12 subunit p35); *IL-23R* (interleukin-23 receptor); *IL-4* (interleukin 4); *JAK2* (Janus kinase 2); *KLF4* (Kruppel-like factor 4); *LIF* (leukemia inhibitory factor); *NLRP3* (NLR family pyrin domain containing 3); *PARP-1* (poly (ADP-ribose) polymerase 1); *RUNX2/HMGB1* (runt-related transcription factor 2/high mobility group box 1); *SIX1* (SIX homeobox 1); *Smad7* (SMAD, family member 7); *SOCS-1* (suppressor of cytokine signaling 1); *SOX6* (SRY-box transcription factor 6); *TLR2* (Toll-like receptor 2); *TLR4* (Toll-like receptor 4); *TSLP* (thymic stromal lymphopoietin); and *YAP1* (Yes-associated protein 1).

Human studies demonstrate that maternal conditions such as asthma and atopy can alter the profile of miRNAs in breast milk (Bozack et al., 2022). More importantly, specific breast milk miRNAs have been identified as protective; for instance, higher infant consumption of miR-148b-3p and miR-375-3p is associated with a reduced risk of the infant developing atopic dermatitis, food allergies, and wheezing (Hicks et al., 2022b).

In asthma, miRNA expression patterns appear highly dependent on clinical activity status. For example, miR-148b-3p was found to be upregulated in the breastmilk of women with inactive asthma (Bozack et al., 2022). Interestingly, in pregnant women, the same milk-derived miRNAs can display inverse expression patterns depending on whether the disease is active or inactive:

Inactive asthma was associated with a general upregulation of milk miRNAs, including miR-193a-5p, miR-224-5p (Bozack et al., 2022), as well as miR-148b-3p, miR-324-5p, and miR-30a-3p (Bozack et al., 2022).

In contrast, active asthma in these patients showed a downregulation of miR-148b-3p, miR-324-5p, and miR-30a-3p, along with other miRNAs such as miR-191-5p and miR-106b-5p (Bozack et al., 2022). A notable exception was miR-1290, which was upregulated in active disease (Bozack et al., 2022).

Collectively, these findings highlight that breast milk miRNA profiles are influenced by maternal atopic status, with expression patterns dependent on the specific condition and its clinical activity.

Table 1 illustrates that certain miRNAs dysregulated in the milk of atopic mothers have established roles in disease pathophysiology; however, the level of evidence supporting these interactions varies significantly.

For multiple candidates, mechanistic data are currently limited to preclinical models. For instance, miR-375-3p targets *YAP1* (Cheng et al., 2020); however, this interaction has been demonstrated only in cell culture studies, lacking direct confirmation in human breast milk or infant samples (Cheng et al., 2020). Similarly, the protective effects of miR-30a-3p against asthma via targets such as *CCR3* (Li X. et al., 2020) rely on evidence from cell and animal models, rather than clinical data.

In contrast to targets validated only in models, some associations are supported by human breast milk expression profiles. *HLA-G*, a gene linked to reduced asthma risk, was identified as a target of miR-148b-3p (Tan et al., 2007). In human milk samples, this miRNA showed a distinct inverse activity pattern–it was upregulated in milk of mothers with inactive asthma but downregulated in mothers with active asthma (Bozack et al., 2022). A similar inverse association was observed for miR-30a-3p (Bozack et al., 2022). Furthermore, many dysregulated milk miRNAs have been characterized in related but distinct atopic conditions using specific cell lines.

A clear example of this is miR-29a-5p, which Table 1 lists as downregulated in active asthma (Bozack et al., 2022) but whose identified target, *FOS,* was validated in the context of allergic rhinitis (Fan et al., 2021), a condition that shares inflammatory pathways with asthma as part of the “united airway disease” concept, yet remains distinct and differs in many aspects of pathophysiology (Klain et al., 2021). Importantly, the interaction was experimentally confirmed in human nasal epithelial cell lines (RPMI2650 and HNEpC), rather than in primary patient cells or blood (Fan et al., 2021).

There is also some evidence for miR-375-3p targets; however in the context of allergic rhinitis. *KLF4* has been validated strictly *in vitro* using human nasal epithelial cells (NECs) (Wang et al., 2020), whereas *JAK2* is supported by evidence from both *in vitro* studies using nasal mucosa cells and *in vivo* murine models involving mouse nasal tissue (Wang et al., 2018). However, no studies have confirmed yet that miR-375-3p targets *JAK2* or *KLF4* within the specific pathophysiology of atopic dermatitis.

Despite these gaps in understanding the specific molecular targets involved, clinical data support a beneficial role of certain miRNAs. Specifically, elevated levels of miR-375-3p in breast milk were statistically associated with a reduced risk of atopic manifestations in infants, including atopic dermatitis, food allergies, and wheezing (Hicks et al., 2022b). To explain this protective potential, *in vitro* studies using human keratinocytes (HaCaT cells) have shown that miR-375-3p may reduce inflammation in atopic dermatitis via the downregulation of *YAP1* (Cheng et al., 2020). Similarly, studies on allergic rhinitis suggest it ameliorates inflammation through the inhibition of *JAK2/STAT3* and *KLF4* signaling (Wang et al., 2018; Wang et al., 2020). However, the mechanism of action of miR-375-3p appears context-dependent. It has also been found to upregulate potential pro-atopic pathways. In nasal epithelial cells, miR-375 was found to upregulate *TSLP*, which in turn stimulates type II innate lymphoid cells (ILC2s) to produce pro-allergic Th2 cytokines (Luo et al., 2020). This suggests that while miR-375-3p has a complex dual function, the overall protective effect of miR-375-3p in certain atopic conditions may result from a predominance of its anti-allergic pathways over its pro-allergic ones.

Interestingly, the study conducted by Simpson et al. also evaluated other miRNAs listed in Table 1, including miR-148b-3p and miR-21-5p. Unlike miR-375-3p, these miRNAs did not show a statistically significant association with reduced atopy risk when assessed individually (Simpson et al., 2015). The authors suggested that the biological effects of altered miRNA expression should be considered collectively, as individual miRNAs are unlikely to play a major role in the development of atopic conditions such as atopic dermatitis (Simpson et al., 2015). Although miR-148b-3p alone was not significantly associated with reduced atopy risk in infants, Hicks et al. reported that higher consumption of this miRNA together with increased miR-375-3p intake enhanced the protective effect of miR-375-3p (Hicks et al., 2022b). In contrast, its interaction with specific *HLA-G* gene genotypes, observed in airway epithelial cells and bronchoalveolar lavage fluid from asthmatic patients, has been linked to asthma development (Tan et al., 2007). These findings indicate that the effects of miR-148b-3p vary depending on the biological context.

In contrast to this complex dual role, other miRNAs like miR-22-3p show a more clearly defined protective function. Simpson et al. identified miR-22-3p as one of the top five most abundant miRNAs in breast milk, noting its association with atopic dermatitis (Simpson et al., 2015). However, functional studies suggest its function is rather beneficial. For instance, Dong et al. discovered that miR-22-3p is directly connected to the pathogenesis of atopic asthma in children, where its downregulation leads to disease exacerbation (Dong et al., 2016). Specifically, significantly reduced levels of miR-22-3p in asthmatic children fail to suppress target genes such as *CBL* and *ESR1*, resulting in their pathological overactivation (Dong et al., 2016). Furthermore, another study identified the *NLRP3* inflammasome gene–a key driver of asthma inflammation through the release of pro-inflammatory cytokines (Guo et al., 2021), as a target notably inhibited by miR-22-3p. Based on these studies, the observed upregulation of miR-22-3p in mothers with atopic dermatitis (Simpson et al., 2015) could have a protective, anti-allergic function intended to transfer immunity to the infant.

Another notably important miRNA is miR-1290, which emerges as a candidate with significant pro-allergic potential. It was the only miRNA among nine studied to be upregulated in association with maternal asthma, showing a positive regression coefficient (B = 2.14), while all other associated miRNAs were downregulated (Bozack et al., 2022). Furthermore, it was also the only miRNA found to be significantly associated with maternal atopy, showing an even stronger positive upregulation (B = 2.38) (Bozack et al., 2022). Hence, miR-1290 may represent a potential factor in breast milk relevant to allergic outcomes. While direct evidence linking miR-1290 to atopic diseases remains limited, studies in other biological contexts have identified miR-1290 targets that are highly relevant to allergic inflammation. For instance, miR-1290 can activate the *JAK/STAT3* and PI3K/AKT pathways by targeting *SOCS4* (Xiao et al., 2018). Additionally, miR-1290 targets *SOCS3*, which regulates Th2-associated M2 macrophage polarization via *STAT3* (Gu et al., 2023). Considering its positive correlation with allergic disease. These indicated mechanisms align with strong clinical upregulation of miR-1290, pointing to a plausible role in enhancing Th2-associated responses.

Overall, these findings underscore the potential role of specific maternal milk miRNAs, such as miR-1290, miR-375-3p, or miR-22-3p, in modulating infant immune development and atopy risk. In addition to peer-reviewed studies, preliminary reports have also pointed to potential roles for miR-155 and miR-21 (Murai et al., 2018) along with a downregulated cluster including miR-342-5p (Prescott et al., 2019) in mothers of infants with atopic dermatitis. However, as these data currently lack formal validation, they were excluded from Table 1. Collectively, these findings highlight the need for further well-controlled studies to confirm the roles of these miRNAs and clarify their mechanistic contributions to infant atopy.

## 4 Discussion

The studies included in this review demonstrate both strengths and limitations. Together, these four studies deepen our understanding of breast milk miRNAs, though they differ in methodology and research focus. Both Bozack et al. and Simpson et al. provided foundational profiling of milk miRNAs. The first used the TaqMan OpenArray platform and validated the study through careful adjustment for key confounders such as race and education (Bozack et al., 2022) whereas Simpson et al. expanded the research by incorporating cytokines and immune cell analyses into miRNA profiling (Simpson et al., 2015). In contrast, Hicks et al. and Ahlberg et al. stand out for their stronger longitudinal study designs. Hicks et al. used quantitative precision to estimate actual miRNA intake rather than relying only on expression levels (Hicks et al., 2022b). Similarly, Ahlberg et al. extended a follow-up to 24 months and linked milk miRNA profiles to specific infant immune markers - Treg levels, shifting the focus from general health outcomes to underlying mechanisms (Ahlberg et al., 2023b).

Despite their strengths, all four studies share fundamental limitations that complicate the ability to draw firm conclusions between specific miRNAs and infant health. A central limitation of the current evidence base is the lack of proof regarding biological uptake. Critically, none of the included studies demonstrated the direct transfer of miRNAs from breast milk into the infant circulation. Consequently, the reported findings rely entirely on statistical associations rather than confirmed methodological pathways. Although the data sources varied, comprising both observational birth cohorts (Bozack et al., 2022; Hicks et al., 2022b) and analyses nested within randomized controlled trials (Simpson et al., 2015; Ahlberg et al., 2023b), the lack of functional validation limits the ability to establish a clear link between maternal milk miRNAs and infant atopy outcomes.

Furthermore, methodological heterogeneity makes direct comparison difficult.

Firstly, the studies investigated miRNAs in different milk fractions, leading to potential profile discrepancies. Bozack et al. isolated EVs (Bozack et al., 2022), Simpson et al. assessed whole milk (Simpson et al., 2015), while both Ahlberg et al. and Hicks et al. focused on the lipid fraction (Ahlberg et al., 2023b; Hicks et al., 2022b). Moreover, the study by Hicks et al. was limited by technical issues regarding exosome isolation (Hicks et al., 2022b), which potentially confused encapsulated miRNAs with other forms due to the study’s focus on the lipid layer.

Secondly, the timing of sample collection varied widely. While Simpson et al. collected samples at a single time point (3 months postpartum) (Simpson et al., 2015), the other studies were longitudinal, sampling at birth and 3 months (Ahlberg et al., 2023b) or across three stages over the first 24 weeks (Hicks et al., 2022b). This discrepancy is significant because miRNA content changes dynamically over time. Finally, the studies faced statistical power limitations due to sample size heterogeneity. While Hicks et al. conducted the largest investigation (n = 163) (Hicks et al., 2022b), Bozack et al. (n = 80) (Bozack et al., 2022) and Simpson et al. (n = 54) (Simpson et al., 2015) were limited by smaller sample sizes.

Although this review provides insights into potential biological mechanisms, the analysis relies on indirect evidence. A key limitation lies in applying cellular data to different disease contexts. As an example, the mechanism for miR-375-3p has been demonstrated only in allergic rhinitis; therefore, these findings cannot be directly applied to atopic dermatitis without specific validation (Wang et al., 2020; Wang et al., 2018). Furthermore, it is critical to distinguish between statistical dysregulation and functional biological relevance. Statistical dysregulation in miRNAs does not necessarily result in altered biological function or cause significant downstream effects on infant physiology. Finally, due to the fact that miRNAs, such as miR-375-3p, affect multiple different pathways–e.g., *YAP1, KLF4, JAK2* (Cheng et al., 2020; Wang et al., 2020; Wang et al., 2018), it is difficult to identify main drivers of atopic diseases.

In summary, while maternal health is associated with distinct breast milk miRNA profiles, the potential link to infant atopy risk remains theoretical. Current evidence is preliminary and limited, hampered by varied study designs and small sample sizes. Consequently, the proposed functions of these miRNAs on the infant often remain hypothetical and contradictory. Therefore, to confirm and establish long-term effects of milk miRNA changes linked to maternal atopy, this field requires larger-scale, prospective cohort studies that monitor atopic status, miRNA expression, and infant health outcomes over many years. This field must also address methodological limitations, such as the lack of standardized protocols for sample collection, processing, and quantification, which make it more difficult to compare results across different studies. Following careful methodological refinement, future studies should test the hypothesis that breast milk miRNA profiles could serve in the future as non-invasive biomarkers for predicting infant allergic disease risk. Furthermore, the potential for maternal interventions, such as dietary modifications, to alter breast milk miRNA composition and prevent atopy remains a theoretical concept requiring validation in controlled clinical trials.

**FIGURE 3:**
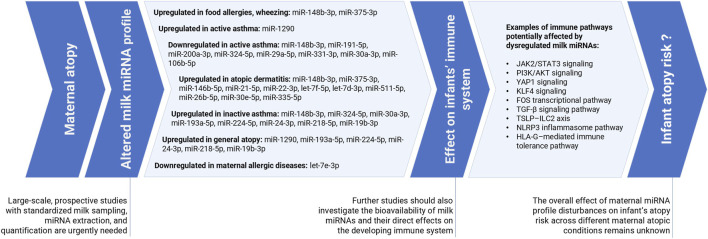
Summary of the findings linking maternal atopy, altered milk miRNA profile and its potential effect on affecting the infants’ immune system and atopy risk with future research directions marked.

Findings linking maternal atopy, altered milk miRNA profiles, and their potential effects on the infant immune system and atopy risk, along with key directions for future research, are summarized in Figure 3.

## 5 Conclusion

In conclusion, this review highlights the link between breast milk miRNA profiles and the risk of infant atopic diseases. Maternal atopy is associated with distinct milk miRNA patterns, such as elevated miR-1290 or miR-375-3p, which may influence infant immune development. Specific miRNAs can modulate both pro- and anti-allergic pathways (Cheng et al., 2020; Lu et al., 2009; Dong et al., 2016), creating a complex regulatory network. However, current evidence is limited by methodological heterogeneity, small sample sizes, and a lack of data on miRNA bioavailability in infants. There is an urgent need for longitudinal, large-scale prospective studies with standardized protocols for milk sampling, miRNA extraction, and quantification. In addition, investigations into the bioavailability of milk miRNAs in infants and their direct, functional effects on the developing immune system and atopy risk are essential to validate these miRNAs as non-invasive biomarkers or potential therapeutic targets.
